# *Bartonella* spp. Bacteremia in Blood Donors from Campinas, Brazil

**DOI:** 10.1371/journal.pntd.0003467

**Published:** 2015-01-15

**Authors:** Luiza Helena Urso Pitassi, Pedro Paulo Vissotto de Paiva Diniz, Diana Gerardi Scorpio, Marina Rovani Drummond, Bruno Grosselli Lania, Maria Lourdes Barjas-Castro, Rovilson Gilioli, Silvia Colombo, Stanley Sowy, Edward B. Breitschwerdt, William L. Nicholson, Paulo Eduardo Neves Ferreira Velho

**Affiliations:** 1 Division of Dermatology, Department of Medicine, State University of Campinas (UNICAMP), Campinas, São Paulo, Brazil; 2 College of Veterinary Medicine, Western University of Health Sciences, Pomona California, United States of America; 3 Department of Molecular & Comparative Pathobiology, Johns Hopkins University School of Medicine, Baltimore, Maryland, United States of America; 4 Centro de Hematologia e Hemoterapia (HEMOCENTRO), Department of Medicine, State University of Campinas (UNICAMP), Campinas, São Paulo, Brazil; 5 Laboratory of Animal Quality Control, Multidisciplinary Center of Biological Investigation (CEMIB), State University of Campinas (UNICAMP), Campinas, São Paulo, Brazil; 6 Department of Virology, Adolfo Lutz Institute (IAL), Secretaria de Estado de Saúde de São Paulo, São Paulo, Brazil; 7 Intracellular Pathogens Research Laboratory, College of Veterinary Medicine, North Carolina State University, Raleigh, North Carolina, United States of America; 8 Rickettsial Zoonoses Branch, Centers for Disease Control and Prevention, Atlanta, Georgia, United States of America; University of Texas Medical Branch, UNITED STATES

## Abstract

*Bartonella* species are blood-borne, re-emerging organisms, capable of causing prolonged infection with diverse disease manifestations, from asymptomatic bacteremia to chronic debilitating disease and death. This pathogen can survive for over a month in stored blood. However, its prevalence among blood donors is unknown, and screening of blood supplies for this pathogen is not routinely performed. We investigated *Bartonella* spp. prevalence in 500 blood donors from Campinas, Brazil, based on a cross-sectional design. Blood samples were inoculated into an enrichment liquid growth medium and sub-inoculated onto blood agar. Liquid culture samples and Gram-negative isolates were tested using a genus specific ITS PCR with amplicons sequenced for species identification. *Bartonella henselae* and *Bartonella quintana* antibodies were assayed by indirect immunofluorescence. *B. henselae* was isolated from six donors (1.2%). Sixteen donors (3.2%) were *Bartonella*-PCR positive after culture in liquid or on solid media, with 15 donors infected with *B. henselae* and one donor infected with *Bartonella clarridgeiae*. Antibodies against *B. henselae* or *B. quintana* were found in 16% and 32% of 500 blood donors, respectively. Serology was not associated with infection, with only three of 16 *Bartonella*-infected subjects seropositive for *B. henselae* or *B. quintana*. *Bartonella* DNA was present in the bloodstream of approximately one out of 30 donors from a major blood bank in South America. Negative serology does not rule out *Bartonella* spp. infection in healthy subjects. Using a combination of liquid and solid cultures, PCR, and DNA sequencing, this study documents for the first time that *Bartonella* spp. bacteremia occurs in asymptomatic blood donors. Our findings support further evaluation of *Bartonella* spp. transmission which can occur through blood transfusions.

## Author Summary

*Bartonella* is a genus of small bacteria with worldwide distribution, transmitted by blood-sucking insects, and is capable of causing disease in humans and animals. Some of the clinical presentations of *Bartonella* spp., such as cat scratch disease, trench fever, and bacillary angiomatosis are well documented; however, novel presentations have been described in the last two decades, ranging from cyclic flu-like syndrome to neurologic disease and life-threatening endocarditis. Asymptomatic human infection is possible and accidental blood transmission has been reported. Bacterium isolation is very difficult because they grow slowly and require special culture media and procedures. Serology testing poorly predicts active *Bartonella* infection, except in infection of cardiac valves. Therefore, diagnosis is generally challenging. However, when molecular detection techniques are coupled with special culture protocols, enhanced sensitivity and specificity can be achieved. We investigated *Bartonella* spp. infection prevalence in a large blood donor population and confirmed bacteremia in 1.2% of the subjects. Bloodstream infection was detected with at least three different molecular methods in 3.2% of donors. These results indicate that *Bartonella* is a genus of importance for transfusion medicine.

## Introduction

*Bartonella*, a genus of fastidious bacteria with worldwide distribution, is responsible for persistent infections in animals and humans [1]. *Bartonella* spp. are considered neglected zoonotic pathogens, presumed to be transmitted to humans by a variety of arthropod vectors including sandflies, body lice, fleas, ticks, and keds [1,2]. During the past several years, the spectrum of clinical manifestations associated with bartonellosis, a term that now encompasses infection with any *Bartonella* spp., has widened substantially [3]. In humans, *Bartonella* spp. are known causative agents of Peruvian bartonellosis, cat scratch disease, trench fever, and bacillary angiomatosis [1]. However, more recent studies have documented bloodstream infections in patients with cardiovascular, neurological, and rheumatologic disease manifestations [4,5]. With the exception of localized lymphadenopathy or blood-culture-negative endocarditis, physicians rarely consider *Bartonella* sp. infection among differential diagnoses [6].

*Bartonella* spp. are able to infect and survive inside erythrocytes [7], resulting in a long-lasting intraerythrocytic and presumably intraendothelial infection, which can be associated with a relapsing pattern of bacteremia [8]. *In vitro*, these bacteria have been shown to invade, multiply within, and persist for the lifetime of the infected host cell [9,10]. Prolonged bacteremia allows greater opportunity for arthropod vector and other modes of transmission to occur between hosts. Although at least fifteen *Bartonella* spp. have been associated with human infections, *B. henselae* is the most frequent species identified from humans, as well as from companion animals such as cats and dogs [1,9]. There is no single gold standard methodology to diagnose bartonellosis and multi-step platforms are necessary to decrease false-negative test results [1]. Culture in liquid and solid media, multiple PCR reactions and serology have been used together to improve the diagnostic sensitivity [8,11].

Previous studies by our group using transmission electron microscopy and culture isolation have documented the ability of *B. henselae* to survive in stored blood for 35 days, suggesting the potential for transfusion-associated transmission [12]. We also documented *B. henselae* adhered to human erythrocytes 10 hours after inoculation of the bacteria into blood and intraerythrocytic infection after 72 hours [13]. These results suggested a potentially important role for *Bartonella* sp. in transfusion medicine, particularly as blood transfusion infection has been documented in cats [14] and needle stick transmission of *Bartonella* sp. has also been reported in two veterinarians [15,16]. Since the presence of selected *Bartonella* spp. was previously documented in blood samples of asymptomatic subjects [17–20], we hypothesized that bloodstream infection with *Bartonella* sp. occurs in blood donors at the time of donation. The objective of this study was to determine the seroprevalence and frequency of bacteremia caused by *Bartonella* spp. in a large asymptomatic blood donor population in Campinas, São Paulo State, Brazil.

## Methods

### Study design and participants

**Figure 1:**
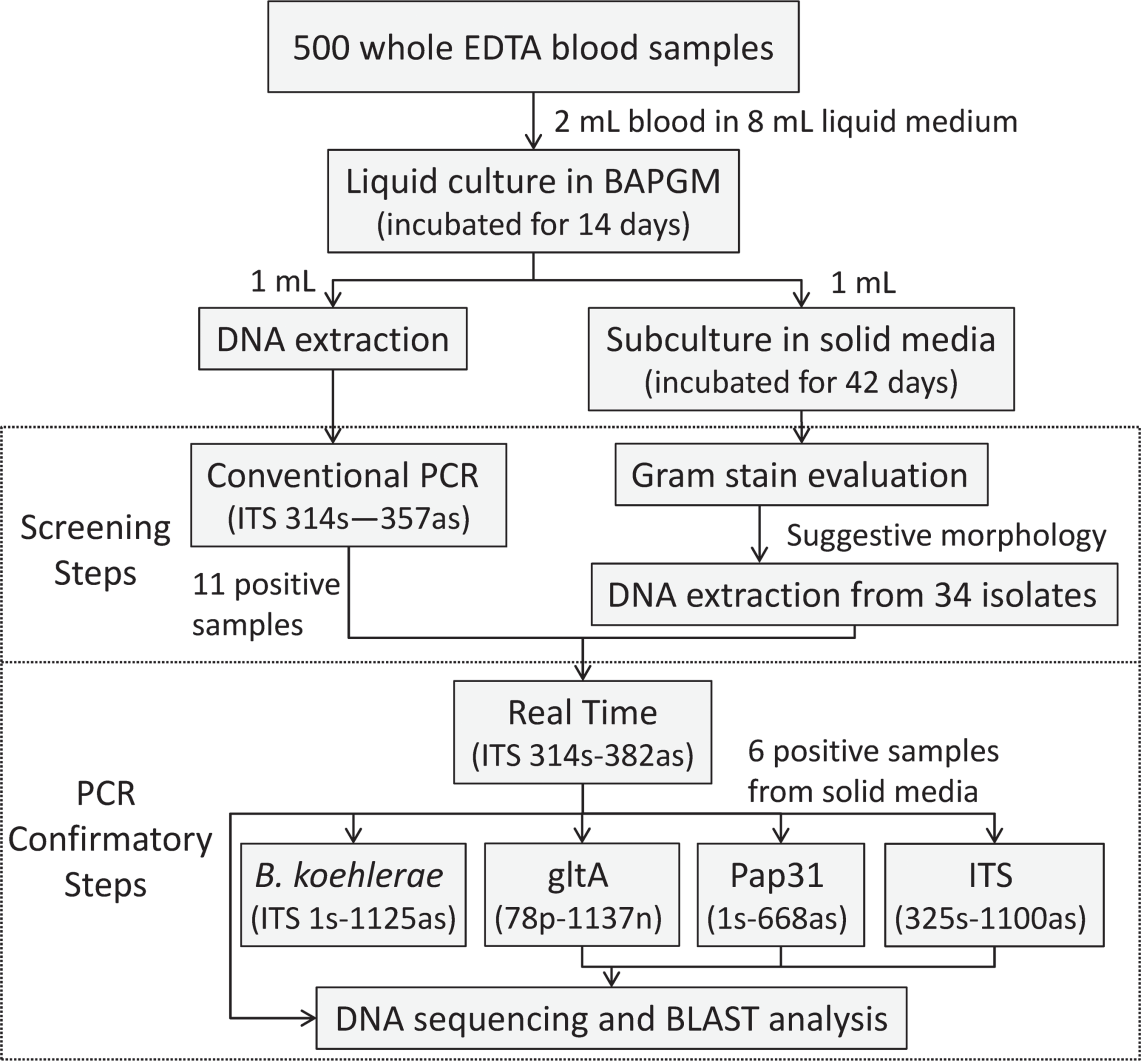
Flowchart of the culture and PCR-based procedures performed to determine *Bartonella* prevalence in 500 blood donors from Campinas, Brazil.

A cross-sectional study was conducted at the UNICAMP Blood Bank (HEMOCENTRO), which serves a geographic region with an estimated population of 6.4 million people in the São Paulo state, Brazil. Healthy blood donors were randomly recruited from November 19^th^ to December 23^rd^ 2009, at the time of their voluntary blood donation. Sample size was estimated in 473 subjects to allow for estimation of at least 5% prevalence of bloodstream infection with *Bartonella* spp. in blood donors, with a desired precision of 5% given a 95% confidence limit and a design effect of 1. Therefore, with possible attrition, we enrolled 500 donors. This study was approved by the Research Ethics Committee of the University of Campinas (UNICAMP), Brazil (CEP122/2005). An informed written consent, approved by the UNICAMP Research Ethics Committee, was obtained from each participant. Donor selection, blood collection, and infectious disease screening were performed in accordance with current international standards [21,22]. Following aseptic preparation of the venipuncture site and immediately after the collection of a blood unit, an additional 5 mL of whole blood was collected into a tube with ethylenediaminetetraacetic acid (EDTA) and another 5 mL was collected into a serum separator tube via the accessory port. Samples were stored at −20°C until analysis at the University of Campinas and subsequently at Western University of Health Sciences. An overview of the diagnostic procedures performed in this study is presented in the Fig. 1.

### Blood culture

Culture procedures used in this study followed the previous description by Duncan *et al*. [11] and Maggi *et al*. [8], with modifications. Two milliliters of whole EDTA blood from each subject were thawed and added into 8 mL of liquid *Bartonella* alpha-Proteobacteria growth medium (BAPGM) [23], incubated at 37°C in 5% CO2, water-saturated atmosphere and maintained with a constant shaking motion for 14 days. Blood donor sample cultures were manipulated in batches of 75 flasks. A negative control flask containing only BAPGM medium was added to each batch of samples tested and subjected to the same laboratory procedures and culture conditions. Subsequently, 1 mL of the blood-inoculated liquid culture was sub-inoculated onto agar enriched slant tubes containing 30% *Bartonella* spp.-negative sheep blood (confirmed by PCR and culture methods) [24] for additional 42 days. BAPGM-negative controls were also subcultured onto blood-agar. The agar slant tubes were inspected weekly for evidence of bacterial growth. Because blood cultures in BAPGM may yield other species of alpha-Proteobacteria [25], an initial screening process was performed as demonstrated in Fig. 1. Colonies were Gram-stained and those isolates with suggestive morphology were suspended and frozen in Brain and Heart Infusion (BHI) for future identification by DNA amplification and sequencing. All *Bartonella* spp. culture methods were carried out in a class 2 biosafety cabinet in order to minimize the possibility of specimen contamination and to protect laboratory personnel.

### *Bartonella* genus PCR screening

Molecular techniques were performed in four separate rooms to avoid DNA contamination. A uni-directional workflow was strictly enforced between pre-PCR areas (sample handling, PCR set up, DNA extraction) and post-PCR areas (DNA amplification, gel analysis, and amplicon purification). Dedicated sets of equipment, pipettes, and supplies were used in each of these locations. Strict laboratory procedures were implemented in order to avoid potential contamination of reagents and samples with amplicons. In order to prevent PCR contamination, different negative controls testing the different stages in the PCR process was included in every experiment as described in the S1 Appendix.

After a 14-day incubation period, a 1 mL aliquot of liquid culture medium was centrifuged and the pellet was subjected to DNA extraction using the QIAamp® DNA Mini Kit (QIAGEN Inc., Valencia, CA). The average of DNA yield obtained was 6.19 ng/ul (SD: 32.8 ng/ul), and the average 260/280 ratio was 1.47 (SD: 2.03). Screening of the 500 liquid culture samples was performed using *Bartonella* genus-specific single tube PCR. This assay was manually designed to target a hypervariable region of the 16S-23S rRNA gene intergenic transcribed spacer (ITS) of *Bartonella* species using primers and conditions described in the S1 Appendix. For all PCR reactions three controls were incorporated: negative BAPGM control (BAPGM medium with no blood inoculate and incubated simultaneously with each batch of liquid cultures), Mastermix reagent control, and a positive control (DNA extracted from a Houston 1-ITS strain of *B. henselae*; ATCC 49882). These screening tests were performed at the Multidisciplinary Center of Biological Investigation (CEMIB), UNICAMP, Brazil.

### *Bartonella* species identification

DNA obtained from *Bartonella* sp. positive liquid cultures and DNA from suspected subculture isolates were retested at Western University of Health Sciences, USA, for species identification. These DNA samples were tested by a Real-Time ITS PCR using primers manually designed to amplify a fragment of the 16S-23S rRNA ITS region of all *Bartonella* species. PCR primers and conditions are described in the S1 Appendix.

DNA samples from six isolates that were confirmed to contain *Bartonella* sp. DNA were further characterized using previously published conventional PCR assays for the ITS region [26], for the citrate synthase gene (*gltA*) [27], and the heme-binding phage-associated protein (*pap*31) gene [26]. In addition, each sample was also tested using a specific conventional PCR for *Bartonella koehlerae* [28]. DNA samples from liquid cultures were not tested by these assays due to insufficient genomic material. Amplicons generated from any one of the five PCR assays used during the confirmatory steps (Fig. 1) were sequenced for bacterial species identification (S1 Appendix).

### Serology

Using *B. henselae* and *B. quintana* antigens supplied by the Centers for Disease Control and Prevention (CDC-Atlanta, USA), serum samples were analyzed for IgG antibodies to *B. henselae* and *B. quintana* antigens by an indirect immunofluorescence assay (IFA) as described in the S1 Appendix. Sera samples were tested at a 1:64 dilution. A positive test was warranted if brightly stained bacteria could be detected by fluorescence microscopy at 400× magnification. Previously IFA negative serum samples were used as negative controls.

### Data analysis

Subjects with positive ITS PCR results from isolates were considered bacteremic. Subjects positive from liquid culture were considered with *Bartonella* sp. bloodstream infection. Molecular and serologic prevalence of *Bartonella* spp. were described as absolute frequencies, percentages, and 95% confidence intervals (computed using score method) using JMP Pro 10 for Windows (SAS Institute Inc., Cary, NC).

## Results

From the 500 blood donors tested, 16 (3.2%, 95% Confidence Interval [CI]: 2.0%–5.1%) subjects were infected with a *Bartonella* spp. based on culture in liquid and/or isolation on solid media followed by ITS Real-Time and/or conventional PCR in two different laboratories. ITS amplicon sequence analysis revealed *B. henselae* in 15 of the 16 cultures (3%, 95% CI: 1.8%–4.9%), and *B. clarridgeiae* in one culture (0.2%, 95% CI: 0%–1.1%) (Table 1). Among the 16 *Bartonella* sp. bloodstream-infected donors, 11 were confirmed by liquid culture followed by PCR amplifications and DNA sequencing, whereas six bacteremic individuals were confirmed after subculture onto blood slant tubes followed by PCR and DNA sequence confirmation (Table 1). Only one subject was PCR positive in both liquid and solid subcultures.

**Table 1:** Serology and DNA sequencing results of 16 blood donors tested for exposure or infection with *Bartonella* species.

**Blood donor**	**Serology (IFA)^a^**		***Bartonella* species identified by DNA sequencing**				
	***B. henselae***	***B. quintana***	**Sample tested**	**ITS^b^ 314s-382as**	**ITS^b^ 325s-1100as**	**Pap31^c^ 1s-668as**	**gltA^d^ 781p-1137n**
1	Negative	Negative	Liquid culture	*B. henselae*	NP^e^	NP	NP
2	Negative	Negative	Liquid culture/isolate	*B. henselae^f^*	*B. henselae*	Negative	Negative
3	Negative	Negative	Isolate	*B. henselae*	Negative	Positive^g^	*B. henselae*
4	Positive	Negative	Liquid culture	*B. henselae*	NP	NP	NP
5	Negative	Negative	Isolate	*B. henselae*	*B. henselae*	*B. henselae*	*B. henselae*
6	Negative	Negative	Isolate	*B. henselae*	Negative	Negative	*B. henselae*
7	Negative	Negative	Isolate	*B. henselae*	*B. henselae*	*B. henselae*	*B. henselae*
8	Negative	Negative	Liquid culture	*B. henselae*	NP	NP	NP
9	Negative	Negative	Liquid culture	*B. henselae*	NP	NP	NP
10	Negative	Negative	Liquid culture	*B. clarridgeiae*	NP	NP	NP
11	Negative	Negative	Liquid culture	*B. henselae*	NP	NP	NP
12	Negative	Negative	Liquid culture	*B. henselae*	NP	NP	NP
13	Negative	Negative	Liquid culture	*B. henselae*	NP	NP	NP
14	Negative	Positive	Liquid culture	*B. henselae*	NP	NP	NP
15	Negative	Negative	Liquid culture	*B. henselae*	NP	NP	NP
16	Positive	Positive	Isolate	*B. henselae*	*B. henselae*	*B. henselae*	*B. henselae*

^a^ Indirect immunofluorescence assay with cut-off of 1:64.

^b^ 16S-23S rRNA gene intergenic transcribed spacer

^c^ Heme-binding phage-associated protein

^d^ Citrate synthase gene

^e^ Not performed due to insufficient genomic material

^f^ Both liquid culture and isolate yield the same *Bartonella* species. Other PCR assays were only performed on isolate due to insufficient genomic material from liquid culture.

^g^ A DNA sequence was not obtained for this sample.

When subsequently amplified and sequenced, four of these six isolates contained a larger ITS region fragment (559 bp in size) that was 100% similar to *B. henselae* sequences deposited in GenBank (accession number NC_005956.1). From one liquid culture sample, a 190 bp ITS DNA sequence was obtained, being 100% similar to *B. clarridgeiae* (accession number NC_014932.1). Similarly, the presence of *B. henselae* DNA was confirmed in five isolates by also amplifying and sequencing the *glt*A gene (338 bp, 100% similarity to accession number BX897699.1), and from three by amplification and sequencing of the *pap*31 gene (501 bp, 100% similarity to accession number DQ529248.1). By testing the isolates, no blood donor was *B. koehlerae* PCR positive using species-specific ITS primers. The methodologies applied in our laboratory allowed the isolation of fastidious bacteria morphologically similar to *Bartonella* by Gram stain from 34 blood donors, with six isolates confirmed as *Bartonella* spp. by DNA sequencing. Some of the possible genera of the other isolates obtained in this study include *Arthrobacter, Bacillus, Dermabacter, Methylobacterium, Propionibacterium, Pseudomonas, Sphingomonas, Staphylococcus* and unknown “non-cultured” bacteria, as previously reported by Cadenas *et al.* using the same liquid enrichment culture method [25].

Of the 500 blood donors tested, antibodies against *B. henselae* or *B. quintana* were detected in 16.2% (81/500, 95% CI: 13.2%–19.7%) and 32% (160/500, 95% CI: 28.0%–36.2%), respectively. Seropositivity for both antigens was detected in 13.4% of blood donors (67/500, 95% CI: 10.6%–16.7%). *B. quintana* DNA was not detected in any blood donor in this study. Only two of the *B. henselae* seroreactive blood donors were confirmed by liquid culture/PCR as infected with *B. henselae*. However, two *B. quintana* seroreactive donors had confirmed *B. henselae* bloodstream infection, one of whom was also seroreactive to *B. henselae*. The *B. clarridgeiae* bacteremic subject was seronegative to both *Bartonella* spp. antigens (Table 1).

## Discussion

Using a combination of liquid and solid cultures, PCR, DNA sequencing, and serology, this study documented the presence of *Bartonella* spp. bloodstream infections in 16 (3.2%) of 500 healthy blood donors presented to a major blood bank in Southeastern Brazil. Despite the fact that exposure of blood donors to *Bartonella* spp. has been previously documented by serology methods [29,30], no previous study has confirmed the presence of *Bartonella* spp. in blood donors using similar culture and molecular diagnostic methods. A total of six *B. henselae* isolates were obtained in this study, with other ten blood donors having blood infection with *B. henselae* or *B. clarridgeiae* identified by liquid culture enrichment coupled with DNA amplification by PCR at two different laboratories. These results indicate, for the first time, that asymptomatic blood donors can be infected with *Bartonella* spp. at the time of blood donation.

*Bartonella* spp. are re-emerging infectious agents that can induce asymptomatic and intraerythrocytic infection in preferred and accidental hosts. These bacteria have been isolated from both immunocompetent and immunodeficient human patients [8,9,31]. Any asymptomatic infection with a blood-borne pathogen has the potential for transfusion transmission. The survival of *B. henselae* in collected human blood units [12] associated with the bacterium’s ability to cause infection by the intravenous route as described in animal models [14,32], and in humans after needle stick accidents [15,16], supports a potential risk for transmission through blood transfusions. Although well-documented human cases of blood transfusion transmission have not yet been published, our data further support the hypothesis that this can occur.

Transmission via blood transfusion is of great relevance if the transfused agent subsequently causes short or long-term disease manifestation in the donor recipient. In humans, cat-scratch fever, bacillary angiomatosis and endocarditis are the most common historically recognized disease entities caused by *Bartonella* sp. infection [1]. However, non-specific symptoms have been described, including fever of unknown origin, local or generalized lymphadenopathy, severe or recurrent anemia, chronic uveitis, optic neuritis, frequent headaches, fatigue, intermittent paresthesias, and granulomatous or angioproliferative reactions of undefined etiologies [15,16,33,34]. Similar non-specific clinical signs have been reported in *Bartonella* sp. bacteremic cats and dogs [35–37].

Diagnosis of bartonellosis remains a microbiological challenge because of the difficulty of culturing and isolating the bacterium from patient specimens. *Bartonella* sp. is highly fastidious and isolation by direct plating of blood or other clinical samples is not always successful. In order to improve the chances of isolation of *Bartonella* sp. from blood, samples should be cultured in liquid enrichment media to support growth [11], similar to culture techniques used for *Haemophilus influenza* and other bacteria species [38]. In our study, *Bartonella* spp. DNA was identified more frequently from BAPGM liquid culture samples than from subculture isolates, with only one subject PCR positive at both culture methods (Table 1). Similar discrepancies between results from liquid and solid culture methods were previously documented. Among 122 *Bartonella*-infected patients in the USA, 48 (39%) had BAPGM liquid culture samples positive for *Bartonella* DNA, but isolates were obtained only from five subjects (4%), including from two patients with PCR-negative liquid culture samples [4]. Causes for these differences are not completely elucidated and may be associated with low levels of bacteremia in asymptomatic humans [1]. In this study, the amount of viable *Bartonella* spp. in the bloodstream of these infected blood donors was not determined. Low bacterial burden in the bloodstream may limit the transmission of *Bartonella* spp. to a blood recipient or the development of infection. In addition, the discrepancy between the number of *Bartonella* sp. isolates obtained and the number of BAPGM samples positive by PCR could indicate that PCR-positive BAPGM samples contained non-viable bacteria. However, the two culture systems used in this study were designed to reproduce the vector environment (insect-based liquid culture medium BAPGM) and the host environment (blood-enriched agar medium), and each culture system may have facilitated the growth and detection of specific wild types of *Bartonella*. Consequently, the use of a combined diagnostic platform for testing the presence of *Bartonella* spp. DNA in BAPGM liquid culture and in subculture isolates in this study provided enhanced sensitivity, as previously demonstrated [39].

Evidence of *Bartonella* sp. infection may be confirmed by microbiological isolation, molecular techniques, and histopathologic visualization of *Bartonella* sp. antigens from tissue samples. Serology can be used to document exposure, but does not confirm infection. In this study, antibodies against *B. quintana* or *B. henselae* were detected in 32% (136/500) and 16.2% (78/500), respectively. A previous Brazilian study involving 437 healthy subjects from a rural area of Minas Gerais state documented *B. quintana* and *B. henselae* seroprevalences of 12.8% and 13.7%, respectively [40]. In Sweden, overall *Bartonella* spp. seroprevalence among 498 blood donors was 16.1%, but only 1.2% of those subjects were seropositive for *B. henselae* [29]. In New Zealand, 5% of 140 blood donors were seropositive for *B. henselae* [30]. In our study, antibodies against *Bartonella* sp. correlated poorly with infection as detected by PCR amplification followed by DNA sequencing or with the successful isolation of *Bartonella* organisms. Similar findings have been previously demonstrated in animals [14,41] and human patients [4,8], even in individuals with symptomatic disease [8,42]. Difficulties in *Bartonella* serodiagnosis are exemplified in the study by Vermeulen *et al.* [42]. It is suggested that *Bartonella* spp. manipulate the host immune system on a systemic scale to achieve a state of immunological attenuation, including stimulation of IL-10 secretion, which suppresses the capabilities of various immune cells, including T helper cells, monocytes/macrophages, and dendritic cells, thus interfering with both innate and adaptive immune responses [9,10]. Therefore, our results indicate that the predictive value of serology to detect *Bartonella* spp. infection in asymptomatic donors is low, supporting the recommendation that antibody status should not be used as a sole diagnostic modality to determine *Bartonella* spp. infection in blood donors. Moreover, negative results in one or more currently available diagnostic tests cannot exclude infection and, whenever possible, a combination of diagnostic tests is encouraged [42,43].

Another factor related to the apparent emergence of *Bartonella* spp. is the development of diagnostic techniques with improved sensitivity [4]. In recent years, the development of more sensitive and specific PCR methods, coupled with enrichment growth in specific culture media, has increased the detection of this pathogen in animal and human patient samples [4,8,11,26]. It is of clinical and epidemiological relevance that failure to amplify *Bartonella* sp. gene targets, following extraction of DNA from patient blood samples, does not rule out this bloodstream infection. It is estimated that *Bartonella* bacteremia in asymptomatic donors is approximately 10 CFU/mL of blood [1], which may be below the detection limit of most conventional or Real- Time PCR assays. Another reason for false-negative PCR or culture results is that *Bartonella* spp. typically cause a cyclic bacteremia [9]. It has been recently demonstrated that the detection of *Bartonella* sp. infection in humans is improved when three sequential blood samples are tested during a one-week period [39]. In that study, only 3 of 12 patients with *Bartonella* sp. bloodstream infection were documented as positive on more than one sample test date and no patient was positive on liquid culture/PCR for all three specimen test dates. Therefore, the number of bacteremic subjects may be underestimated in our study because only one blood sample from each blood donor was tested. We hypothesize that the actual number of blood donors infected with a *Bartonella* spp. may be higher in healthy humans than our current findings have documented. The low bacterial levels and the cyclic feature of *Bartonella* sp. bloodstream infection reinforce this hypothesis [9,10].

Based on recommendations by the Ethics Committee from UNICAMP Medical School, *Bartonella* sp. positive blood donors in this study were considered inappropriate for further blood donations. To the authors’ knowledge, there are no specific guidelines in the USA or other countries designed to prevent transfusion of human blood when donors are suspected to be infected with a *Bartonella* species. In Veterinary Medicine, at least two major medical boards have issued recommendations regarding blood donors. In 2005, the American College of Veterinary Internal Medicine (ACVIM) conditionally recommended the screening of canine and feline blood donors in order to obtain a *Bartonella*-free donor pool, especially for cats due to the high frequency of bacteremia in this host [44]. This recommendation for cats has been recently ratified by the European Advisory Board on Cat Diseases (ABCD) [45]. The results of our study indicate that guidelines for human blood transfusions should be designed, with special attention to the selection of *Bartonella*-free blood products for transfusion to immune suppressed subjects, which would include pediatric and geriatric patients.

The results of this study indicate that human exposure to *Bartonella* spp. frequently occurs in the Southeast region of Brazil, and that *Bartonella* sp. bacteremia occurs in asymptomatic blood donors. There is a risk of blood supply contamination with these pathogens from asymptomatic bacteremic donors. The impact of transmission of *Bartonella* spp. through blood transfusions recipients should be evaluated, as well as the use of advanced diagnostic techniques for the screening of *Bartonella* sp. infection among blood donors.

## Supporting Information

S1 Appendix Detailed protocol of PCR assays used for DNA amplification and identification of *Bartonella* spp, as well as detection of specific antibodies against *Bartonella henselae* and *B. quintana*. (DOCX)

S1 Checklist STROBE Checklist. (DOC)
