# Supplementary material for: Bartonella spp. Bacteremia in Blood Donors from Campinas, Brazil
Source: PLoS Negl Trop Dis. 2015 Jan 15;9(1):e0003467. doi: 10.1371/journal.pntd.0003467 (PMC4295888; doi:10.1371/journal.pntd.0003467)
Supplement: S1 Appendix — (DOCX) [file pntd.0003467.s001.docx]

**Appendix S1**

**Table of Contents**

Table S1. Primers used in this study 2

Supplementary methods 3

PCR conditions 3

*Bartonella* species identification 4

Serology 4

Supplementary References 5

**Table S1: Primers used in this study.**

| Application | Target gene | Primer designation | Sequence (5’-3’) | Length of amplicon (bp) | Reference |
| --- | --- | --- | --- | --- | --- |
| *Bartonella* spp.  Conventional PCR | 16S-23S ITS | 314A-s  314B-s  357-as | CGTTTCTCTTTCTTCMGATGATGATCCC  CGTTTCTCTTTCTTCRGATGATGATCCC  GCGCTCTAACCAACTGAGC | 157 - 271 | This study |
|  |  | 325-s  1100-as | CTTCAGATGATGATCCCAAGCCTTYTGGCG  GAACCGACGACCCCCTGCTTGCAAAGCA | 408 - 673 | 1 |
|  | *gltA* gene | BhCS.781p  BhCS.1137n | GGGGACCAGCTCATGGTGG  AATGCAAAAAGAACAGTAAACA | 379 | 2 |
|  | *pap31* gene | 1s  668as | ACTTCTGTTATCGCTTTGATTTCRRCT  CACCACCAGCAAAATAAGGCATMAY | 526 - 557 | 1 |
| *B. koehlerae*  Conventional PCR | 16S-23S ITS | 1s  1125as | CTTCTAAAATATCGCTTCTAAAAATTGGCATGC  GCCTTTTTTGGTGACAAGCACTTTTCTTAAG | 527 | 3 |
| *Bartonella* spp.  Real-time PCR | 16S-23S ITS | 314A-s  314B-s  382-as | CGTTTCTCTTTCTTCMGATGATGATCCC CGTTTCTCTTTCTTCRGATGATGATCCC  ACTTSAACCTCCGACCTCACGCTTAT | 190 - 304 | This study |

**Methods S1**

***PCR conditions***

The conventional PCR targeting the 16S-23S rRNA gene intergenic transcribed spacer (ITS) of *Bartonella* species was performed on a 25 μL reaction mixture containing 0.25 μL of Taq DNA polymerase (5U per μL), 0.4 μΜ of each primer (314A-s, 314B-s and 357-as) and 5 μL of DNA template. Amplifications were performed under the following conditions: one hot-start cycle at 95⁰C for 2 minutes followed by 45 cycles of denaturing at 95⁰C for 60 seconds, annealing at 58⁰C for 60 seconds, and extension at 72⁰C for 30 seconds. Amplification was completed by an additional cycle at 72⁰C for 5 minutes, and products were analyzed by 2% agarose gel electrophoresis under UV exposure. A known concentration of *B. henselae* DNA was serially diluted 10-fold from 10^9^ to 1 genome equivalent (GE) per microliter to determine the sensitivity of the PCR assay. The sensitivity of this assay was established at a minimum of 50 genome equivalent (GE) of *B. henselae* per reaction tube.

The real-time PCR targeting the 16S-23S rRNA ITS of *Bartonella* species was performed on a 25μl reaction mixture contained 1X PCR mix (SYBR^®^ Premix Ex Taq, Takara Bio Inc., Shiga, Japan), 7.5 pmol of each primer 314A-s and 314B-s, 10.5 pmol of primer 382-as, 1X of ROX reference dye, and 5 μl of DNA template . Amplifications were performed under the following conditions: one hot-start cycle at 95⁰C for 1 minute followed by 50 cycles of denaturing at 94⁰C for 10 seconds, annealing at 58⁰C for 20 seconds, and extension at 72⁰C for 20 seconds. Amplification was detected by SYBR Green I fluorescence with emission at 522 nm. Melting curves were obtained for each amplification. A limit of detection of 5 copies per reaction was determined using the approach described above.

***Bartonella* species identification**

Amplicons generated from any one of the five PCR assays used during the confirmatory steps were purified (MiniElute kit, Qiagen, Valencia, California, USA) and sequenced with a fluorescence-based automated sequencing system (Eurofins MWG Operon, Huntsville, AL, USA). Chromatogram evaluation, primer deletion and sequence alignment were performed using Contig Express and AlignX softwares (Vector NTI Suite 10.1, Invitrogen Corp., Carlsbad, CA, USA). *Bartonella* spp. were defined by comparing similarities with other sequences deposited in the GenBank database using the Basic Local Alignment Search Tool (BLAST) [4].

**Serology**

Using CDC protocol, IFA testing was performed using Vero cell culture grown antigens of *B. henselae*, and *B. quintana,* according to a previously described procedure [5] and positive controls were obtained from positive patients with *Bartonella* sp. infection. Serum samples were diluted 1/64 in phosphate-buffered saline (PBS), and 25 μL was applied to *Bartonella*-infected cells fixed to a glass slide and incubated for 30 min at 37°C. The slides were washed three times (5 minutes each) in PBS, overlaid with fluorescein isothiocyanate-conjugated goat antihuman immunoglobulin IgG (Sigma, St. Louis, MO, USA) at a dilution of 1/150, incubated at 37^o^ C for 30 minutes, washed and dried. Slides were then mounted and read on a fluorescent microscope. The cutoff titer of the IFA was 1/64. Endpoint titers were not determined.

**References S1**

1. Diniz PP, Maggi RG, Schwartz DS, et al. (2007) Canine bartonellosis: serological and molecular prevalence in Brazil and evidence of co-infection with *Bartonella henselae* and *Bartonella vinsonii* subsp. *berkhoffii*. Vet Res 38:697-710.
2. Norman AF, Regnery R, Jameson P, Greene C, Krause DC. (1995) Differentiation of *Bartonella*-like isolates at the species level by PCR-restriction fragment length polymorphism in the citrate synthase gene. J Clin Microbiol 33:1797-803.
3. Varanat M, Maggi RG, Linder KE, Breitschwerdt EB. (2011) Molecular prevalence of *Bartonella*, *Babesia*, and hemotropic *Mycoplasma* sp. in dogs with splenic disease. J Vet Intern Med 25:1284-91.
4. Altschul SF, Gish W, Miller W, Myers EW, Lipman DJ. (1990) Basic local alignment search tool. J Mol Biol 215:403-10.
